# Supplementary material for: Expression of Elafin and CD200 as Immune Checkpoint Molecules Involved in Celiac Disease
Source: Int J Mol Sci. 2024 Jan 10;25(2):852. doi: 10.3390/ijms25020852 (PMC10815464; doi:10.3390/ijms25020852)
Supplement: Supplementary file 1 [file ijms-25-00852-s001.zip › ijms-2769149-supplementary.pdf]

Supplemantary materials

Supplementary Table S1. Clinical data of patients with Celiac disease

Patient	Age (year)	Sex	AATG (IgA)	AAEM	Atrophy grade (Marsh criteria)	HLA-DQB1
**Celiac 1**	4	F	>200	+	3C	0201-0202
**Celiac 2**	4	F	>200	+	3C	0201-0202
**Celiac 3**	1	F	>150	+	3B	0301-0302
**Celiac 4**	3	F	>50	+	3A	0201-0603
**Celiac 5**	12	M	>200	+	3C	0201-0503
**Celiac 6**	7	M	>125	+	3A	0201-0301
**Celiac 7**	1	F	>125	+	2	0201-0602
**Celiac 8**	5	F	>125	+	3A	0201-0501
**Celiac 9**	10	M	>200	+	3C	0201-0301
**Celiac 10**	2	F	>125	+	3B	0301-0302
**Celiac 11**	10	F	>125	+	3B	0201-0202
**Celiac 12**	2	F	>25	+	1	0301-0302
**Celiac 13**	3	M	>125	+	3C	0201-0604
**Celiac 14**	5	M	>200	+	3C	0201-0202
**Celiac 15**	6	F	>150	+	3A	0201-0501
**Celiac 16**	8	F	>125	+	3A	0201-0503
**Celiac 17**	2	M	>125	+	3B	0201-0301
**Celiac 18**	11	F	>200	+	3C	0201-0202
**Celiac 19**	7	M	>200	+	3C	0201-0501
**Celiac 20**	9	F	>125	+	3B	0201-0301
**Celiac 21**	6	F	>150	+	3A	0201-0301
**Celiac 22**	12	F	>150	+	3C	0201-0202
**Celiac 23**	7	M	>200	+	3C	0201-0202
**Celiac 24**	10	F	>50	+	3B	0201-0503
**Celiac 25**	11	F	>200	+	3C	0301-0302
**Celiac 26**	6	M	>125	+	2	0201-0602
**Celiac 27**	9	F	>200	+	3B	0201-0501
**Celiac 28**	3	M	>200	+	3A	0301-0302
**Celiac 29**	5	F	>125	+	3A	0201-0301
**Celiac 30**	10	M	>150	+	3B	0201-0202
**Celiac 31**	13	F	>50	+	2	0201-0603
**Celiac 32**	5	M	>150	+	3C	0301-0302
**Celiac 33**	3	F	>200	+	3C	0201-0301
**Celiac 34**	4	M	>150	+	3C	0201-0501
**Celiac 35**	6	M	>200	+	3B	0201-0202
**Celiac 36**	4	F	>125	+	3B	0201,0501
**Celiac 37**	2	F	>150	+	3C	0303,0601
**Celiac 38**	2	F	>150	+	3C	0201,0202
**Celiac 39**	6	F	>200	+	3C	0201,0501
**Celiac 40**	1	M	>50	+	2	0302,0301
**Celiac 41**	12	F	>200	+	3C	0201,0301
**Celiac 42**	2	F	>125	+	3	0201,0202
**Celiac 43**	8	F	>200	+	3C	0202,0301
**Celiac 44**	1	F	>200	+	3C	0201,0202
**Celiac 45**	5	M	>125	+	3B	0301,0302

**Celiac 46**	10	M	>150	+	3C	0202,0301
**Celiac 47**	2	F	>50	+	1	0201/0101
**Celiac 48**	7	F	>150	+	3B	0201/0202
**Celiac 49**	2	F	>50	+	2	0201/0202
**Celiac 50**	1	F	>125	+	3A	0201/0202
**Celiac 51**	2	M	>200	+	3C	0201/0202
**Celiac 52**	2	M	>150	+	3B	0301/0202
**Celiac 53**	3	M	>50	+	2	0502/0602
**Celiac 55**	1	F	>125	+	3C	0602/0604
**Celiac 56**	7	F	>100	+	3C	0201/0503
**Celiac 57**	7	M	>125	+	3A	0201/0303
**Celiac 58**	2	F	>150	+	3B	0201/0301

AAEM: antiendomysial antibody; AATG: antitransglutaminase antibody, expressed as U/ml; HLA: human leukocyte antigen
